# Predicting the potential distribution of *Atractylodes lancea* in China under CMIP6 climate scenarios using an optimized MaxEnt model

**DOI:** 10.3389/fpls.2026.1881018

**Published:** 2026-08-03

**Authors:** Yunyun Sun, Jinchuan Guo, Shimao Chen, Xiaosu Xiang, Wanbo Xing, Yan Zhang, Qiya Ji, Yunan Cao, Tangyi Peng, Qingshan Yang

**Affiliations:** 1 College of Pharmacy, Anhui University of Chinese Medicine, Hefei, China; 2 The First Affiliated Hospital of Anhui University of Chinese Medicine (Anhui Provincial Hospital of Traditional Chinese Medicine), Hefei, China; 3 Anhui Province Key Laboratory of Research & Development of Chinese Medicine, Hefei, China; 4 Institute of Conservation and Development of Traditional Chinese Medicine Resources, Anhui Academy of Chinese Medicine, Hefei, China

**Keywords:** *Atractylodes lancea*, climate change, environmental variables, maxent model, potential habitat prediction

## Abstract

*Atractylodes lancea* is an important medicinal plant in China, and climate change may affect wild-resource conservation and cultivation planning. However, its current potential ecological suitability, future range dynamics, and relationship with traditional or reported producing regions remain insufficiently clarified under optimized species distribution modelling and CMIP6 climate scenarios. We used 220 occurrence records and environmental variables related to climate, topography, vegetation, and soil to predict the current and future potential suitable habitats of *A. lancea* in China with an optimized MaxEnt species distribution model. The final parameterization (feature class = LH; regularization multiplier = 3) was selected using partial ROC, the 5% training omission rate, and AICc. The optimized model retained good discrimination against background locations (mean training AUC = 0.923) while reducing the omission rate and AICc relative to the default settings. July precipitation, temperature seasonality, altitude, and vegetation subtype were identified as the main environmental drivers. Under current conditions, suitable habitats were mainly distributed in northern, central, and eastern China, covering approximately 2.72 × 10^6^ km^2^. Under SSP1-2.6 and SSP5-8.5, projections based on the selected BCC-CSM2-MR climate model indicated a general increase in total suitable area, while geographic centroid locations fluctuated among western Shandong, southern Hebei, and northern Henan. The county/district-level overlay showed that several modern or reported producing regions in Hubei and Henan overlapped well with predicted suitable habitats, whereas some Maoshan-related traditional producing regions showed relatively weak current climatic suitability. These results suggest that historical Dao-di (authentic provenance) production regions of *A. lancea* are not determined solely by present-day climatic suitability, but also reflect historical distribution, cultivation traditions, harvesting and processing practices, and quality evaluation systems. Because the model used one GCM and did not incorporate dynamic land use, harvesting pressure, cultivation management, or medicinal-quality indicators, the results should be interpreted as potential ecological suitability rather than as direct evidence of actual resource distribution, cultivation yield, or geoherbal quality.

## 1 Introduction

Anthropogenic climate forcing has been highlighted by successive IPCC assessments as a major source of pressure on terrestrial vegetation (Anderson, 2013), with cascading effects on phenology, productivity and the geographical ranges of plant species. Such effects are particularly relevant for medicinal plants, whose growth is often constrained by relatively narrow climatic envelopes; range contractions or shifts can not only reduce the available wild stock but also alter the secondary-metabolite profile, and therefore the medicinal quality, of the harvested material (Applequist et al., 2020; Rana et al., 2020). Quantitatively delineating the future ranges of medicinal species is thus a prerequisite for designing wild-resource protection strategies, for guiding the spatial layout of artificial cultivation, and for safeguarding the supply chain of traditional Chinese medicinal materials.

A range of correlative species distribution models, such as BIOCLIM, DOMAIN, GARP, GAM, GLM, ENFA, and MaxEnt, have been developed to address questions of this kind. Among them, the maximum entropy approach implemented in MaxEnt has gained particular traction for medicinal-plant work (Elith et al., 2011; Phillips et al., 2006), because it can be fitted reliably from presence-only records, captures non-linear responses to environmental gradients, and tends to retain reasonable accuracy even when occurrence sample sizes are limited. The method has been applied to growth zoning and climate-response assessments for many medicinal species, including Panax notoginseng (Zhan et al., 2022) and other medicinal plants (Yang et al., 2013). Even so, MaxEnt outputs are sensitive to user-controlled parameters, and tuning the feature class (FC) and regularization multiplier (RM) to suppress overfitting has therefore become an active line of methodological refinement (Merow et al., 2013).

*Atractylodes lancea* (Thunb.) DC., a perennial herb of the genus *Atractylodes* (Asteraceae) (Wu et al., 2011), enters the pharmacopoeia through its dried rhizomes, which are recognized in traditional Chinese medicine for drying dampness, fortifying the spleen, dispelling wind-cold and improving vision. The rhizomes accumulate sesquiterpenoid constituents, with atractylon, atractylodin, beta-eudesmol and related atractylenolides being prominent, and these compounds have been associated with anti-inflammatory, gastroprotective, hepatoprotective and antitumor activities (Bailly, 2021; Jun et al., 2018; Koonrungsesomboon et al., 2014). Because demand for *A. lancea* medicinal material has increased while wild resources remain spatially uneven, identifying climatically and ecologically suitable regions is important for resource conservation and cultivation planning.

Previous studies have provided useful information on the ecological suitability, quality regionalization, and producing-region characteristics of *A. lancea* (Zhu et al., 2017; Wang et al., 2023a; Zhang et al., 2023). Nevertheless, several gaps remain. Some studies have focused mainly on current or historical suitability, leaving the spatial dynamics of suitable habitats under CMIP6 scenarios less fully examined; some have not explicitly evaluated how MaxEnt feature classes and regularization multipliers affect model complexity and overfitting risk; and the county/district-level relationship between traditional or reported producing regions and modelled current suitability has rarely been visualized directly. Clarifying these points is important because predicted ecological suitability, historical Dao-di recognition, and medicinal-material quality do not necessarily represent the same spatial process.

Accordingly, this study used *A. lancea* across mainland China as the research object and tested four working hypotheses: (1) precipitation and temperature-seasonality variables would be the dominant environmental constraints on the potential distribution of *A. lancea*; (2) tuning the MaxEnt feature class and regularization multiplier would reduce omission error and model complexity relative to the default setting while retaining high discrimination ability; (3) suitable-habitat area, spatial pattern, and geographic centroid positions would change under SSP1-2.6 and SSP5-8.5; and (4) traditional or reported producing regions would show only partial overlap with predicted current ecological suitability at the county/district scale. By combining optimized MaxEnt prediction, CMIP6 scenario projection, centroid migration analysis, and producing-region overlay, the study aims to provide a spatial reference for *A. lancea* resource conservation and production planning without equating modelled ecological suitability with realized resource abundance or Dao-di medicinal-material quality.

## 2 Materials and methods

### 2.1 Collection and processing of distribution data for *A. lancea*

Occurrence records of *A. lancea* were compiled from three open-access biodiversity portals: the Chinese Virtual Herbarium (CVH, https://www.cvh.ac.cn/), the National Specimen Information Infrastructure (NSII, http://www.nsii.org.cn/), and the Global Biodiversity Information Facility (GBIF, https://www.gbif.org/). To align temporally with the climate baseline, only records dated 1980 onwards were retained. Records without coordinates, duplicated coordinates, vague locality descriptions, or localities that could not be reliably georeferenced were excluded. For specimens that carried clear locality names but no coordinates, latitude and longitude were supplemented using Google Earth only after checking that the locality description was sufficiently precise. Records were excluded if their reported coordinate uncertainty exceeded 5 km, or if their locality descriptions could not be georeferenced to within approximately 5 km; this threshold matched the spatial thinning distance and was close to the working spatial scale of the resampled environmental layers. The initial compilation produced 264 raw records.

**Figure 1:**
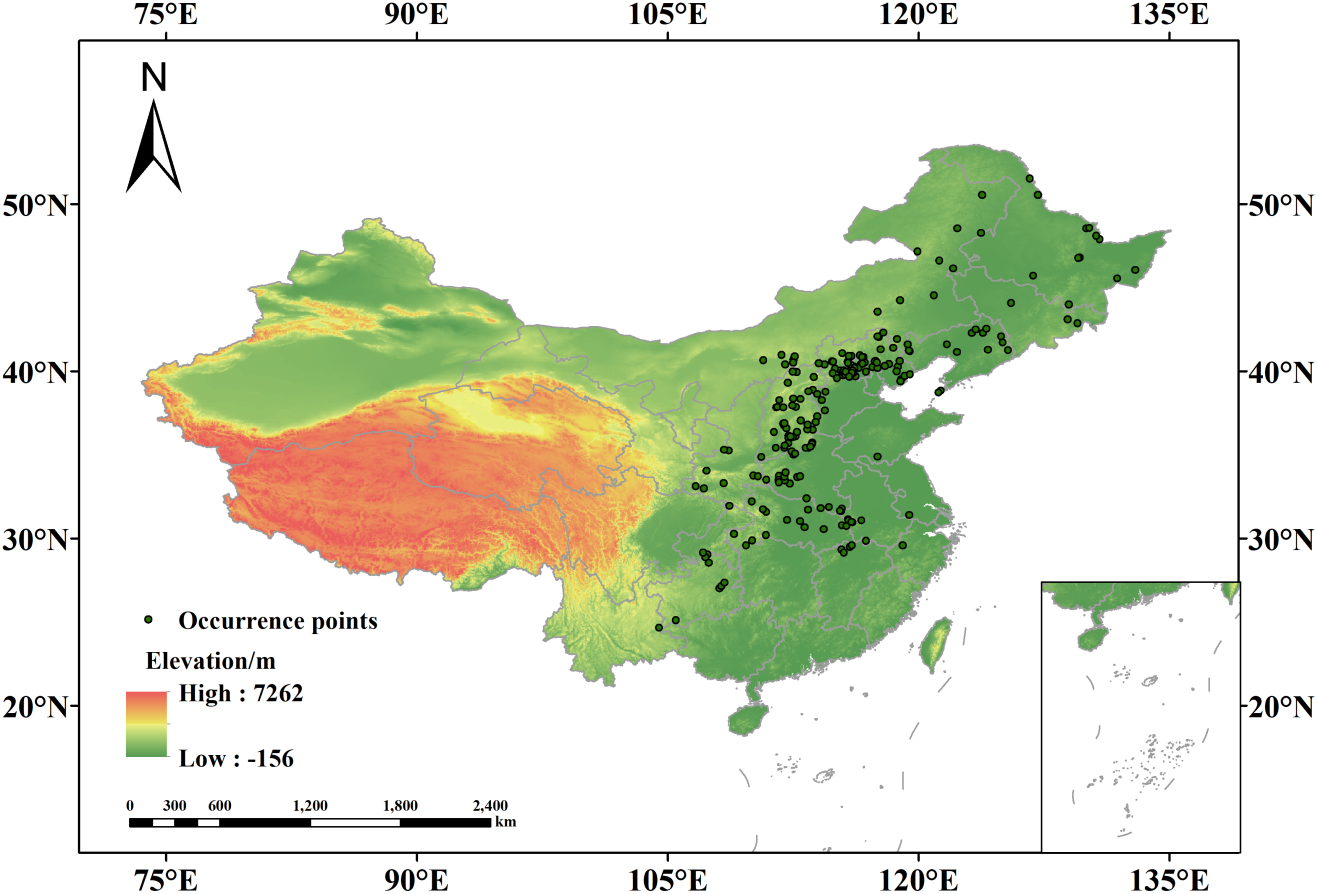
Spatial distribution of *A. lancea* occurrence points in China.

To reduce spatial clustering and local sampling aggregation, occurrence records were filtered using a 5 km × 5 km grid (Boria et al., 2014), with one occurrence retained per grid cell and duplicate or closely spaced records removed. The 5 km thinning distance is close to the spatial scale of the environmental layers after resampling to 2.5 arc-min resolution, and therefore reduces repeated occurrence points within the same or adjacent environmental grid cells. Filtering yielded 220 valid occurrence points, which were saved as a three-column CSV file (species, longitude, and latitude) and used for model calibration and validation. Their spatial distribution is shown in **Figure 1**.

### 2.2 Acquisition and processing of environmental variables

A pool of 90 candidate environmental variables was assembled, covering climate, topography, vegetation, and soil. Climate layers for both the present and future periods came from the WorldClim database (Hijmans et al., 2005; available at https://worldclim.org/) and comprised the 19 standard bioclimatic variables (bio_1-bio_19) plus monthly precipitation and monthly maximum, minimum, and mean temperatures from January through December. Terrain variables, including altitude, slope, and aspect, were derived from a digital elevation model (DEM). Vegetation subtype was included as a vegetation-related predictor, and soil texture and physicochemical attributes were obtained from the Harmonized World Soil Database (HWSD). All layers were resampled to a uniform 2.5 arc-min grid to maintain spatial consistency across data sources. Detailed information for the 90 variables is provided in **Table 1**.

**Table 1:** Detailed information on the 90 environmental variables.

Variable code	Environmental factor	Variable code	Environmental factor
prec 1-12	January to December precipitation	alt	Altitude
tavg 1-12	January to December average temperature	slope	Slope
tmax 1-12	January to December maximum temperature	aspect	Aspect
tmin 1-12	January to December minimum temperature	zbyl	Vegetation Classification
bio1	Annual Mean Temperature	coarse	Coarse fragments
bio2	Mean Diurnal Range	sand	Sand
bio3	Isothermality	slit	Slit
bio4	Temperature Seasonality	clay	Clay
bio5	Max Temperature of Warmest Month	bulk	Bulk Density
bio6	Min Temperature of Coldest Month	ref_bulk	Reference Bulk Density
bio7	Temperature Annual Range	org_cbn	Organic Carbon Content
bio8	Mean Temperature of Wettest Quarter	ph	pH in water
bio9	Mean Temperature of Driest Quarter	n	Total nitrogen content
bio10	Mean Temperature of Warmest Quarter	cn	Carbon/Nitrogen ratio (C/N)
bio11	Mean Temperature of Coldest Quarter	cec_soil	CEC soil
bio12	Annual Precipitation	cec_clay	CEC clay
bio13	Precipitation of Wettest Month	teb	TEB
bio14	Precipitation of Driest Month	bsat	Base Saturation
bio15	Precipitation Seasonality	alum_sat	Aluminium saturation
bio16	Precipitation of Wettest Quarter	esp	Exchangeable Sodium Percentage
bio17	Precipitation of Driest Quarter	eq	Calcium Carbonate
bio18	Precipitation of Warmest Quarter	gypsum	Gypsum content
bio19	Precipitation of Coldest Quarter	elec_con	Electric Conductivity

Future climate fields were drawn from the BCC-CSM2-MR model in CMIP6 (Eyring et al., 2016; Wu et al., 2019), evaluated for four 20-year windows centered on the 2030s (2021-2040), the 2050s (2041-2060), the 2070s (2061-2080), and the 2090s (2081-2100). BCC-CSM2-MR was used as a representative CMIP6 model developed by the Beijing Climate Center and commonly applied in East Asian and China-focused climate-impact studies. Two contrasting shared socioeconomic pathways were used: SSP1-2.6, representing a low-emission, sustainability-oriented future, and SSP5-8.5, representing a high-emission, fossil-fuel-intensive future (Riahi et al., 2017). Because only one GCM was used, the future maps and area statistics should be interpreted as projections under the selected GCM and emission scenarios rather than as a full multi-model climate-uncertainty ensemble.

**Figure 2:**
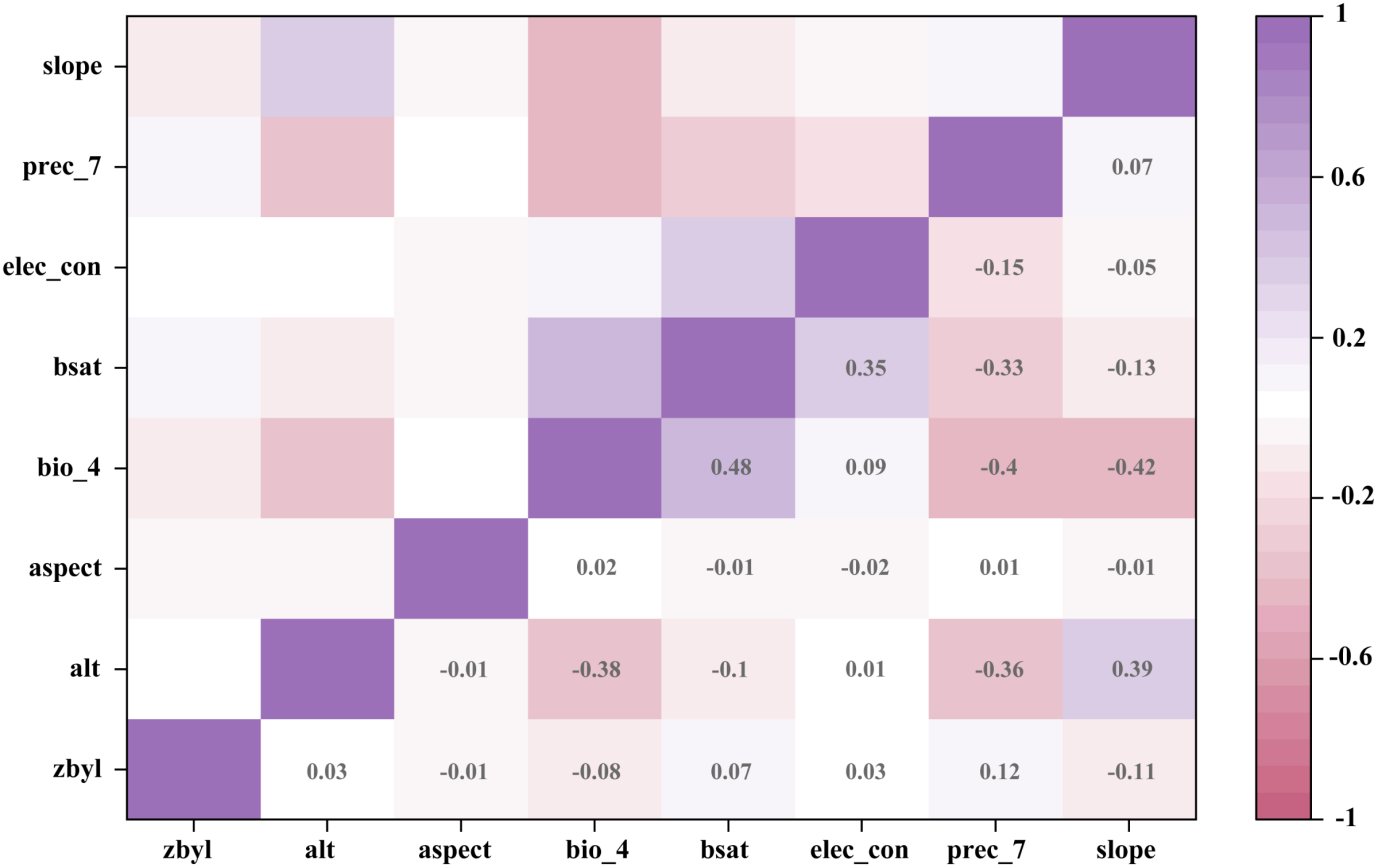
Pearson correlation matrix of the eight retained environmental variables.

Strong collinearity among environmental layers can increase model complexity and reduce the stability of ecological niche models (Dormann et al., 2013). Candidate predictors were therefore screened by combining correlation analysis, ecological interpretability, auxiliary contribution information, and VIF verification. Pearson correlation coefficients were first calculated in ENMTools for all candidate pairs. For each pair with |r| >= 0.8, the variable with clearer ecological meaning and data interpretability was preferentially retained; preliminary MaxEnt percent contribution was used only as supporting information when ecological interpretations were comparable, not as an independent deletion or retention rule. Variance inflation factor (VIF) analysis was then used to verify the final predictor set. The eight retained predictors were July precipitation (prec_07), temperature seasonality (bio_4), altitude (alt), vegetation subtype (zbyl), slope, base saturation (bsat), aspect, and electrical conductivity (elec_con). Their recalculated VIF values ranged from 1.001 to 2.270, all below the commonly used threshold of 5, indicating that severe multicollinearity was not present in the final variable set. The Pearson correlation matrix for the eight retained environmental variables is presented in **Figure 2**.

### 2.3 Construction, parameter optimization and accuracy assessment of the MaxEnt model

The 220 occurrence points and the eight retained environmental variables were imported into MaxEnt 3.4.4 to construct the species distribution model. Run settings were as follows: random partitioning of the data, with 75% used for training and 25% reserved for testing; bootstrap resampling; a maximum of 10,000 background points; the random-seed option enabled to ensure reproducibility; 10 replicate runs; and logistic output.

MaxEnt offers five feature classes: linear (L), quadratic (Q), product (P), threshold (T), and hinge (H). Because both feature combination (FC) and regularization multiplier (RM) affect model complexity and transferability, default settings may lead to overfitting if they are used without evaluation (Phillips et al., 2006; Warren and Seifert, 2011; Merow et al., 2013). We therefore used the kuenm workflow in R to tune model parameters systematically (Muscarella et al., 2014; Cobos et al., 2019). The five feature classes generated 31 candidate FC schemes, and RM values were scanned from 0.5 to 5.0 at intervals of 0.5, producing 310 candidate parameter combinations. Candidate models were evaluated jointly using partial ROC, the 5% training omission rate, and AICc. The final model used FC = LH and RM = 3 because this combination met the statistical and omission-rate requirements while maintaining the lowest AICc among the acceptable candidates.

Model accuracy was evaluated using the area under the receiver operating characteristic (ROC) curve (AUC). The mean training AUC reported by MaxEnt was used consistently when comparing the default and optimized parameter settings. Mean training AUCs range from 0 to 1 and describe the model’s ability to discriminate occurrence locations from background locations. Values of 0.5-0.6 indicate failure, 0.6-0.7 poor performance, 0.7-0.8 fair performance, 0.8-0.9 good performance, and values above 0.9 excellent performance. Because training AUC alone does not measure transferability, model selection followed the kuenm framework and jointly considered partial ROC, the 5% training omission rate, and AICc. To provide an additional threshold-dependent validation metric, the true skill statistic (TSS) was calculated at the MTSPS threshold as sensitivity + specificity - 1. Sensitivity was derived from the independent test occurrence records, whereas specificity was estimated from the background/grid cells classified as unsuitable at the same threshold. TSS values range from -1 to 1, with values closer to 1 indicating better discrimination.

### 2.4 Suitability classification and analysis of spatiotemporal range dynamics

To visualize the predicted habitats of *A. lancea* under both present and future climates, the mean of the 10 MaxEnt replicate outputs (in ASC format) was loaded into ArcGIS 10.8.1. Suitability classes were delineated with the Maximum Test Sensitivity Plus Specificity (MTSPS) threshold (Liu et al., 2005), which jointly accounts for sensitivity and specificity, is reported directly by MaxEnt and is generally more ecologically meaningful than the natural-breaks alternative. The MTSPS value obtained here was 0.2727. Using the Reclassify tool in ArcGIS, the study area was partitioned into four classes: unsuitable (0–0.2727), low-suitability (0.2727–0.5), medium-suitability (0.5–0.7) and high-suitability (20.7–1.0); the area of each class was then derived from raster cell counts.

For the spatial-pattern analysis under future scenarios, the same MTSPS threshold was applied to collapse the predicted surface into two classes only — suitable (MTSPS–1.0) versus unsuitable (0–MTSPS). The Intersect tool of ArcGIS was used to overlay the present-day suitable surface with each future scenario, and the resulting cells were labelled as expansion, contraction, or retention (no-change) areas.

The geographic centroid of the suitable surface was used to represent the overall spatial position of *A. lancea* in each period. Assuming that the species has some intrinsic dispersal capacity and ignoring interspecific interactions, centroid coordinates under the present climate and each future scenario were calculated with the Zonal Geometry tool in ArcGIS (Yang et al., 2022). Connecting the centroids of consecutive periods produced vector trajectories that characterized the direction and distance of range displacement under climate change.

It should be noted that the model constructed in this study represents potential ecological suitability based on climatic, topographic, vegetation-related, and soil-related environmental variables. Therefore, the model results reflect environmental constraints on the potential distribution of *A. lancea*, but they should not be interpreted as the actual distribution of resources or as directly available cultivation areas. Actual production and resource conservation are also affected by land use, human harvesting pressure, cultivation management, market demand, conservation policies, and medicinal-material quality evaluation.

## 3 Results

### 3.1 Model parameter optimization and accuracy evaluation

In a MaxEnt run, the omission rate, mean AUC ratio, and corrected Akaike Information Criterion (AICc) are principal indicators used to balance goodness of fit against model complexity. Under the default settings (FC = LQPH, RM = 1), AICc was 5195.49, the omission rate was 5.4%, the mean AUC ratio was 1.6915, and the mean training AUC was 0.930 (SD = 0.005). Although the mean training AUC was high, the higher omission rate and AICc indicated that the default model was less parsimonious.

**Figure 3:**
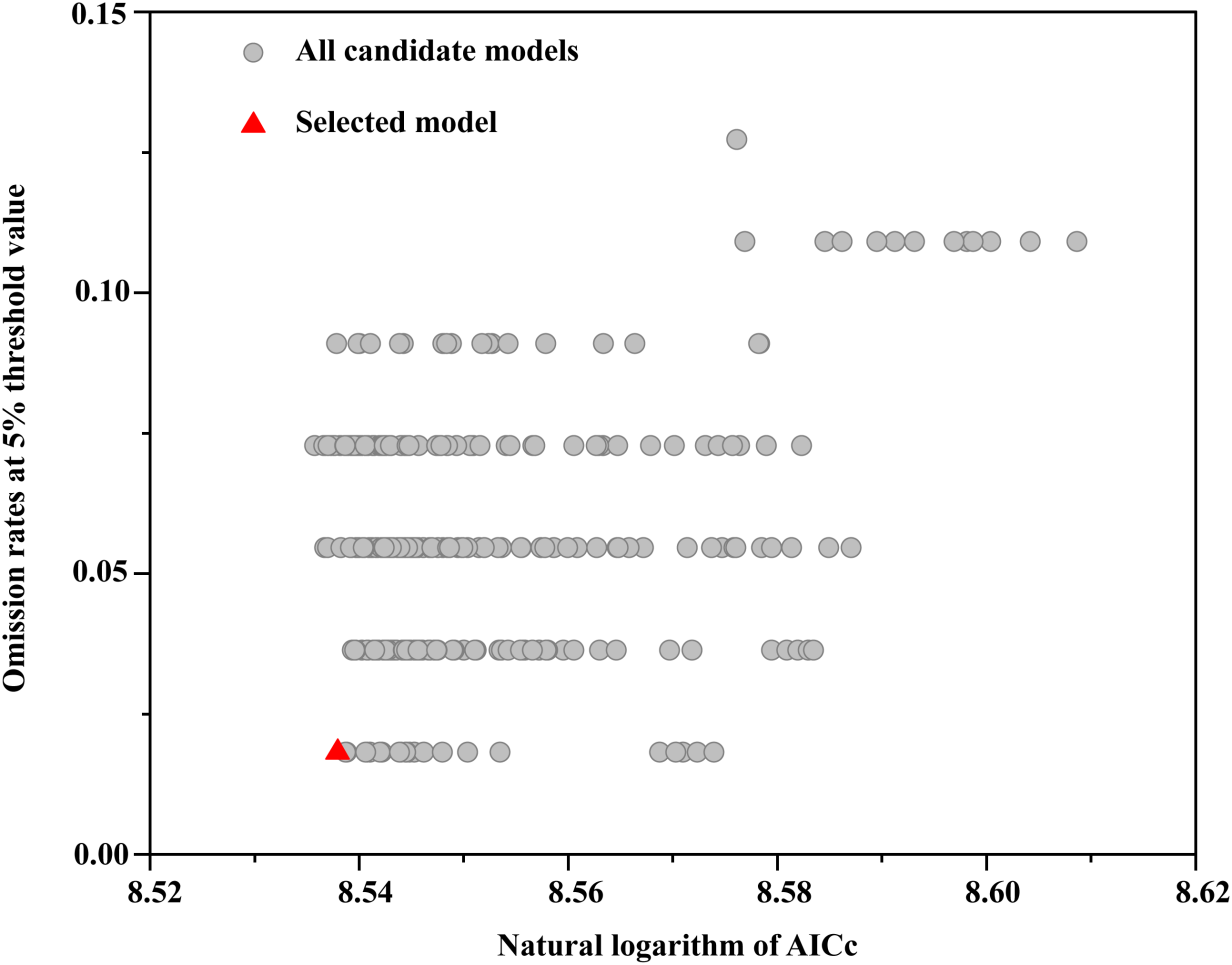
MaxEnt model parameter optimization results.

The kuenm package was used to scan the 310 parameterizations produced by crossing 31 FC schemes with 10 RM levels, and the optimal combination was identified as FC = LH (linear plus hinge features) with RM = 3. Under this combination, the model was statistically significant (p < 0.05) with delta AICc = 0; the omission rate decreased to 1.8%, AICc to 5104.89, and the mean AUC ratio was 1.6012, while the mean training AUC was 0.923 (SD = 0.007). At the MTSPS threshold (0.2727), the optimized model had a test sensitivity of 0.982 (54 of 55 independent test presences correctly classified), a background-based specificity of 0.718, and a TSS of 0.700. The parameter-tuning surface is shown in **Figure 3**, and the default and optimized models are compared in **Table 2**. Their mean training ROC curves are provided in **Supplementary Figure 1**.

**Table 2:** Performance evaluation of the MaxEnt model under the default and the optimized parameter settings.

Model evaluation	Feature combination	Regularization multiplier	Omission rate (%)	Mean AUC ratio	AICc	AUC value
Default	LQPH	1	5.4	1.6915	5195.49	0.930
Optimized	LH	3	1.8	1.6012	5104.89	0.923

The mean training AUC of the optimized model (0.923) was slightly lower than that of the default model (0.930), whereas the omission rate decreased from 5.4% to 1.8% and AICc also declined. These changes indicate a better balance between fit and complexity, supporting the use of the optimized parameterization for the subsequent projections without interpreting the small AUC difference alone as evidence of improved transferability.

### 3.2 Key environmental variables influencing the distribution of *A. lancea*

The percent contribution and permutation importance of the eight retained variables, as estimated by MaxEnt for the distribution of *A. lancea*, are summarized in **Table 3**. July precipitation (prec_07) and temperature seasonality (bio_4) emerged as the two most influential predictors, with percent contributions of 38.5% and 23.2% and permutation importance values of 48.9% and 28.8% respectively, jointly accounting for 61.7% of the total contribution. Altitude (alt, 16.0%) and vegetation subtype (zbyl, 14.7%) followed, and together these four variables explained 92.4% of the modelled distribution, identifying them as the principal environmental drivers of *A. lancea*. The remaining four variables (slope, base saturation, aspect and electrical conductivity) collectively contributed only 7.5%, indicating a comparatively weak control on the spatial pattern.

**Table 3:** Percent contribution and permutation importance of the eight retained environmental variables.

Variable code	Environmental factor	Unit	Percent contribution/%	Permutation importance/%
prec_07	Precipitation in July	mm	38.5	48.9
bio_4	Temperature Seasonality	°C × 100	23.2	28.8
alt	Altitude	m	16	14.9
zbyl	Vegetation Classification		14.7	2
slope	Slope	°	5	3.7
bsat	Base Saturation	%	1.6	0.9
aspect	Aspect	rad	0.7	0.4
elec_con	Electric conductivity	dS/m	0.2	0.4

**Figure 4:**
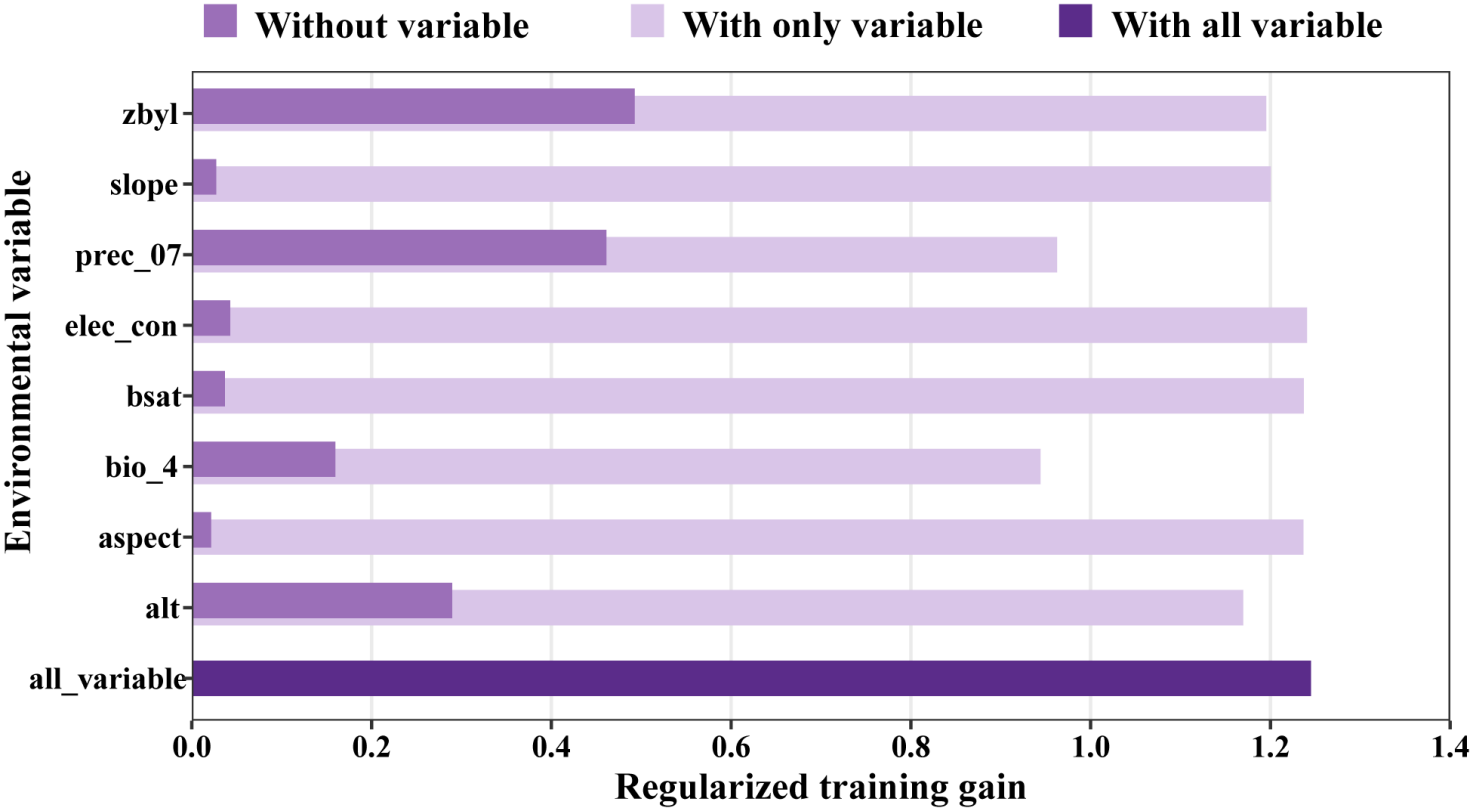
Results of the jackknife test for the importance of the environmental variables in MaxEnt.

A complementary jackknife test (**Figure 4**) reinforced the same ranking. When fitted alone, prec_07 and bio_4 returned the two highest regularized training gains, which means they each carry the greatest stand-alone explanatory power for the distribution of *A. lancea*. Taken together, the contribution-based and the jackknife-based evidence both single out July precipitation and temperature seasonality as the principal climatic drivers of the distribution of *A. lancea* in China.

**Figure 5:**
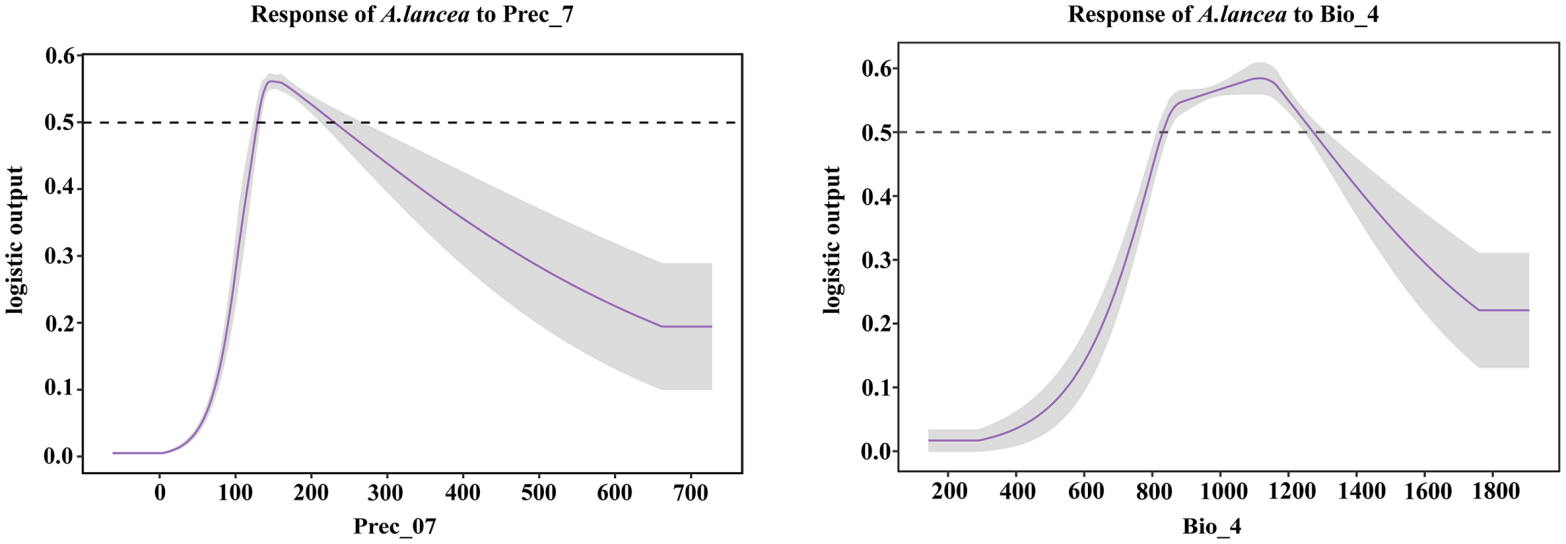
Response curves of the dominant environmental variables (prec_07, bio_4) for the suitable habitat of *A. lancea.*.

Response curves of the dominant predictors (**Figure 5**) further describe the ecological tolerance of *A. lancea*. Environmental values associated with a logistic output above 0.5 were treated as favorable. For July precipitation, the response was unimodal: occurrence probability was close to zero below about 80 mm, exceeded 0.5 within 129–229 mm, peaked near 146 mm at approximately 0.54, and declined above 229 mm, indicating that excessive summer rainfall is unfavorable for *A. lancea*. The response to temperature seasonality (bio_4) was also unimodal, with a peak occurrence probability of approximately 0.58; values that were either too low or too high were unfavorable for the species. These relatively narrow favorable ranges indicate that *A. lancea* depends on a specific combination of moisture and temperature conditions.

### 3.3 Current suitable habitat pattern of *A. lancea*

**Figure 6:**
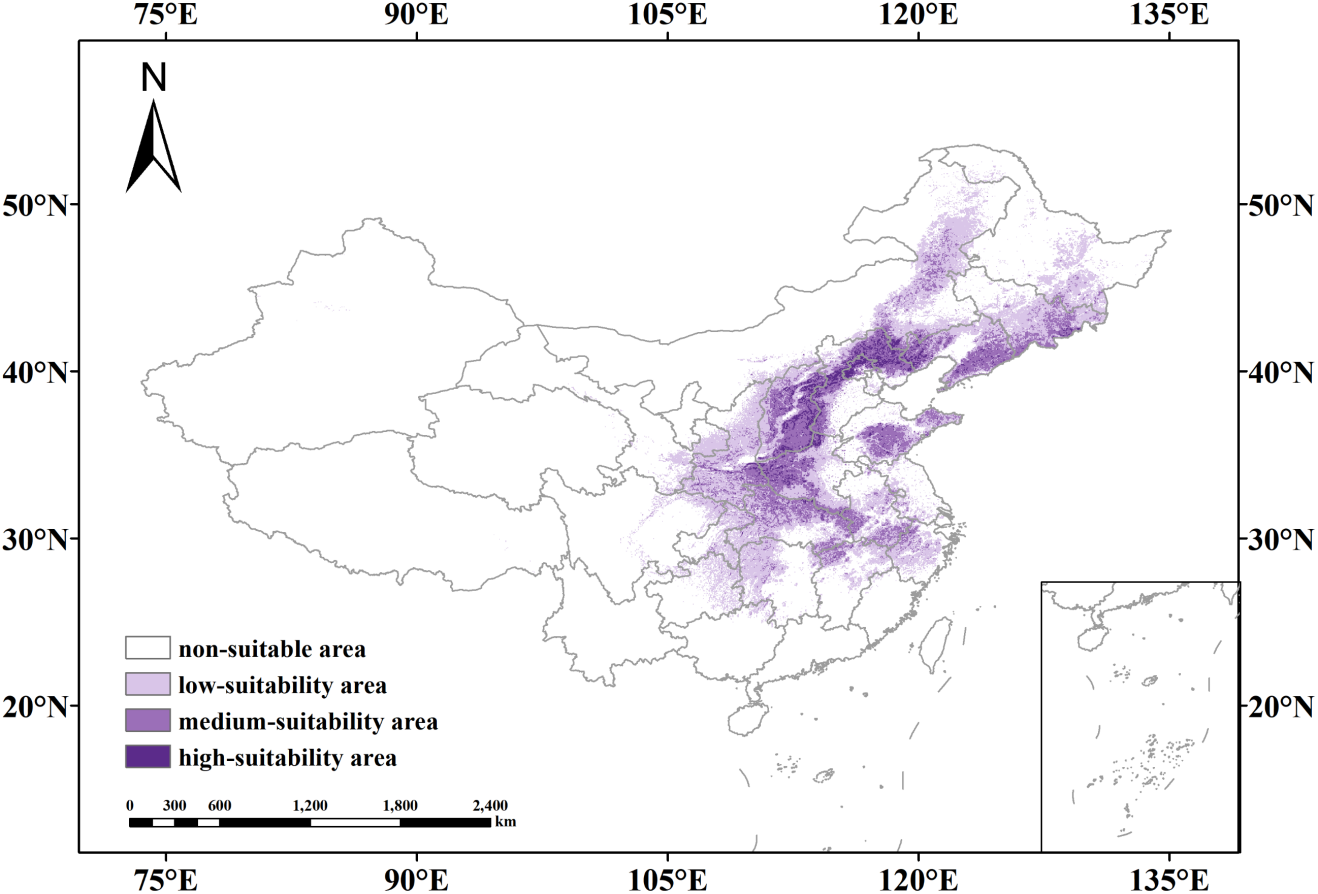
Potential suitable distribution of *A. lancea* under the present climate.

Drawing on the optimized MaxEnt output and using MTSPS = 0.2727 as the suitability threshold, the present-day potential habitat of *A. lancea* across China was reclassified, and the area of each suitability class was tallied in ArcGIS 10.8.1 (**Figure 6**).

Across China the total suitable habitat amounts to 2.72 × 10^6^ km^2^ under the present climate, equivalent to 28.21% of the territory. Of this, the high-suitability class (0.7–1.0) covers 1.40 × 10^5^ km^2^ (1.45% of national land area), centered on western Liaoning, the northern part of the Shandong Peninsula, the northern North China Plain and northern Henan, where favorable hydro-thermal conditions sustain wild populations and support the production of high-quality medicinal material; the medium-suitability class (0.5–0.7) covers 7.81 × 10^5^ km^2^ (8.12%), wrapping around the high-suitability core and extending across western Henan, southeastern Shanxi, central-southern Hebei, most of Shandong, southeastern Shaanxi, northern Hubei and the southern Anhui mountains; the low-suitability class (0.2727–0.5) covers 1.79 × 10^6^ km^2^ (18.64%), forming a belt around the higher-suitability cores that reaches westward into eastern Gansu and northern Sichuan, southward into northeastern Guizhou and northern Guangxi, and northward into eastern Inner Mongolia and southern Heilongjiang. The unsuitable area accounts for 6.91 × 10^6^ km^2^ and is distributed mainly across arid northwestern China, the alpine Qinghai-Tibet Plateau, the Yunnan-Guizhou Plateau, southern China and northern Northeast China.

Spatially, the suitable habitat of *A. lancea* is largely concentrated in the warm-temperate monsoon zone of northern China and follows a roughly concentric pattern, with the North China Plain at the core.

### 3.4 County/district-level producing regions and current predicted suitability

**Figure 7:**
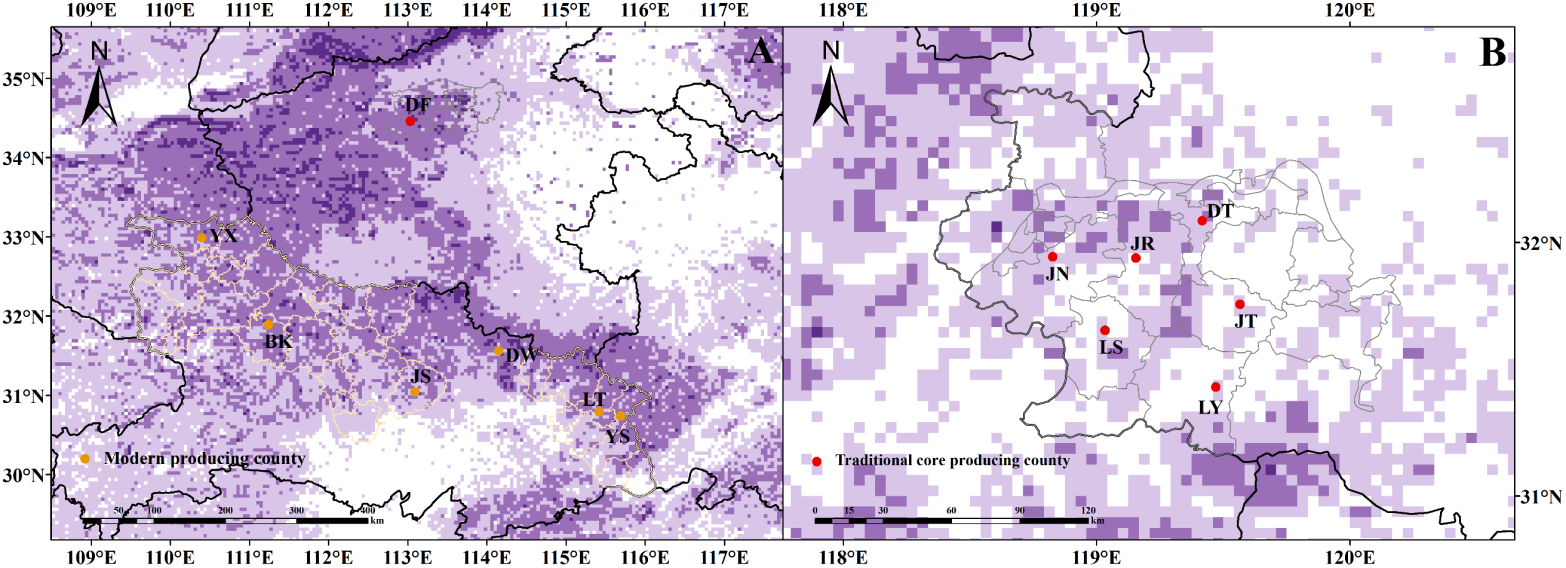
County-level producing regions and current predicted suitability of *A. lancea*. YX, Yunxi; BK, Baokang; JS, Jingshan; DW, Dawu; LT, Luotian; YS, Yingshan; DF, Dengfeng; JN, Jiangning; LS, Lishui; JR, Jurong; DT, Dantu; LY, Liyang; JT, Jintan. [**(A)** Central China producing areas; **(B)** Maoshan and nearby traditional areas].

To further evaluate the relationship between predicted current suitability and traditional or reported producing regions of *A. lancea*, we compiled county- or district-level producing regions from the literature and public sources and overlaid them with the predicted current suitability map (**Figure 7**). The results showed clear regional differences in the degree of overlap. Several modern or reported producing counties in Hubei and Henan overlapped well with predicted suitable habitats. For example, Yingshan, Luotian, Yunxi, Baokang, Jingshan, Dawu, and Dengfeng all contained large proportions of low-, medium-, or high-suitability habitats, and Dawu and Dengfeng showed particularly high proportions of medium- to high-suitability area.

In contrast, several Maoshan-related traditional producing regions in Jiangsu, including Jurong, Dantu, Jiangning, Jintan, Liyang, and Lishui, generally showed weaker current climatic suitability in the model, with only local overlap with low- or medium-suitability habitats. These results indicate that predicted current ecological suitability was consistent with some modern or reported producing regions, but did not fully match all traditional Dao-di medicinal-material production regions. This pattern provides a basis for discussing the formation of Dao-di medicinal-material production regions, historical changes in producing areas, and the influence of non-climatic factors.

The current suitability classes were derived from the optimized MaxEnt model using the thresholds adopted in this study. Literature-reported traditional/geoherbal core counties or districts and modern/reported producing counties were overlaid at the county/district scale. This analysis was used to evaluate the general spatial consistency between reported producing regions and modelled current ecological suitability, rather than to identify township-level production sites.

### 3.5 Suitable distribution of *A. lancea* under future climate scenarios

**Figure 8:**
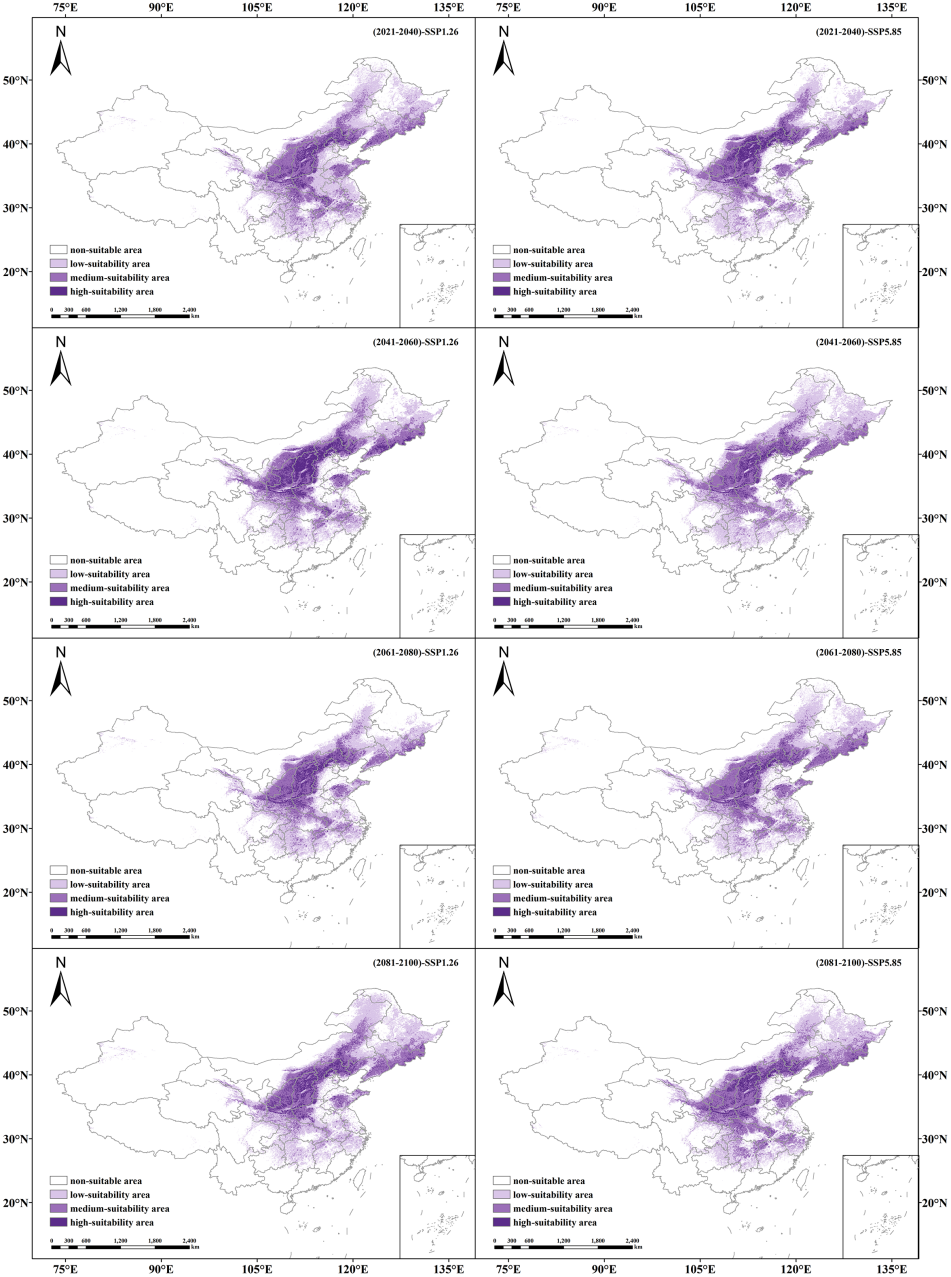
Suitable habitat distribution of *A. lancea* under future climate scenarios (SSP1-2.6 and SSP5-8.5).

Using BCC-CSM2-MR climate fields under CMIP6, potential habitats of *A. lancea* were projected for the 2030s through the 2090s under both SSP1-2.6 and SSP5-8.5. The areas of each suitability class are summarized in **Table 4**, while the corresponding spatial distributions are shown in **Figure 8**.

**Table 4:** Area of suitable habitats for *A. lancea* under current and future climate scenarios by suitability levels.

Decade scenarios	Predicted area (× 10^4^ km^2^)				
	Low habitat suitability	Medium habitat suitability	High habitat suitability	Unsuitable habitat	Total suitable area
Current	179.44	78.14	13.95	690.82	271.53
2030s-SSP1-2.6	233.22	142.46	32.45	554.22	408.13
2050s-SSP1-2.6	191.39	134.12	60.76	576.08	386.27
2070s-SSP1-2.6	187.77	121.27	31.91	621.4	340.95
2090s-SSP1-2.6	223.55	126.96	28.87	582.97	379.38
2030s-SSP5-8.5	174.32	129.64	40.03	618.36	343.99
2050s-SSP5-8.5	218.15	146.81	30.86	566.53	395.82
2070s-SSP5-8.5	200.24	151.69	31.30	579.12	383.23
2090s-SSP5-8.5	194.71	158.77	40.73	568.14	394.21

Under the SSP1-2.6 low-emission pathway, the total suitable area for *A. lancea* follows a fluctuating expansion-then-partial-retreat trajectory. It reaches its maximum of 4.08 × 10^6^ km^2^ in the 2030s and then settles at 3.86 × 10^6^ km^2^ in the 2050s — still 1.15 × 10^6^ km^2^ above the present-day baseline; in parallel, the high-suitability class peaks at 6.08 × 10^5^ km^2^. The total suitable area then drops to 3.41 × 10^6^ km^2^ in the 2070s, and although it rebounds to 3.79 × 10^6^ km^2^ by the 2090s, the high-suitability class itself contracts to 2.89 × 10^5^ km^2^.

Under the SSP5-8.5 high-emission pathway, the total suitable area for *A. lancea* follows an overall steady increase relative to the present climate. It rises from 3.44 × 10^6^ km^2^ in the 2030s (a gain of 7.25 × 10^5^ km^2^ over the present), to 3.96 × 10^6^ km^2^ in the 2050s (gain of 1.24 × 10^6^ km^2^), to 3.83 × 10^6^ km^2^ in the 2070s (gain of 1.12 × 10^6^ km^2^), and finally to 3.94 × 10^6^ km^2^ in the 2090s (gain of 1.23 × 10^6^ km^2^). Within this trajectory the high-suitability class also rises overall and reaches 4.07 × 10^5^ km^2^ by the 2090s.

Combining the two pathways, the total suitable habitat for *A. lancea* shows an overall expanding tendency relative to the present climate.

### 3.6 Spatial-pattern changes in the future potential distribution of *A. lancea*

**Figure 9:**
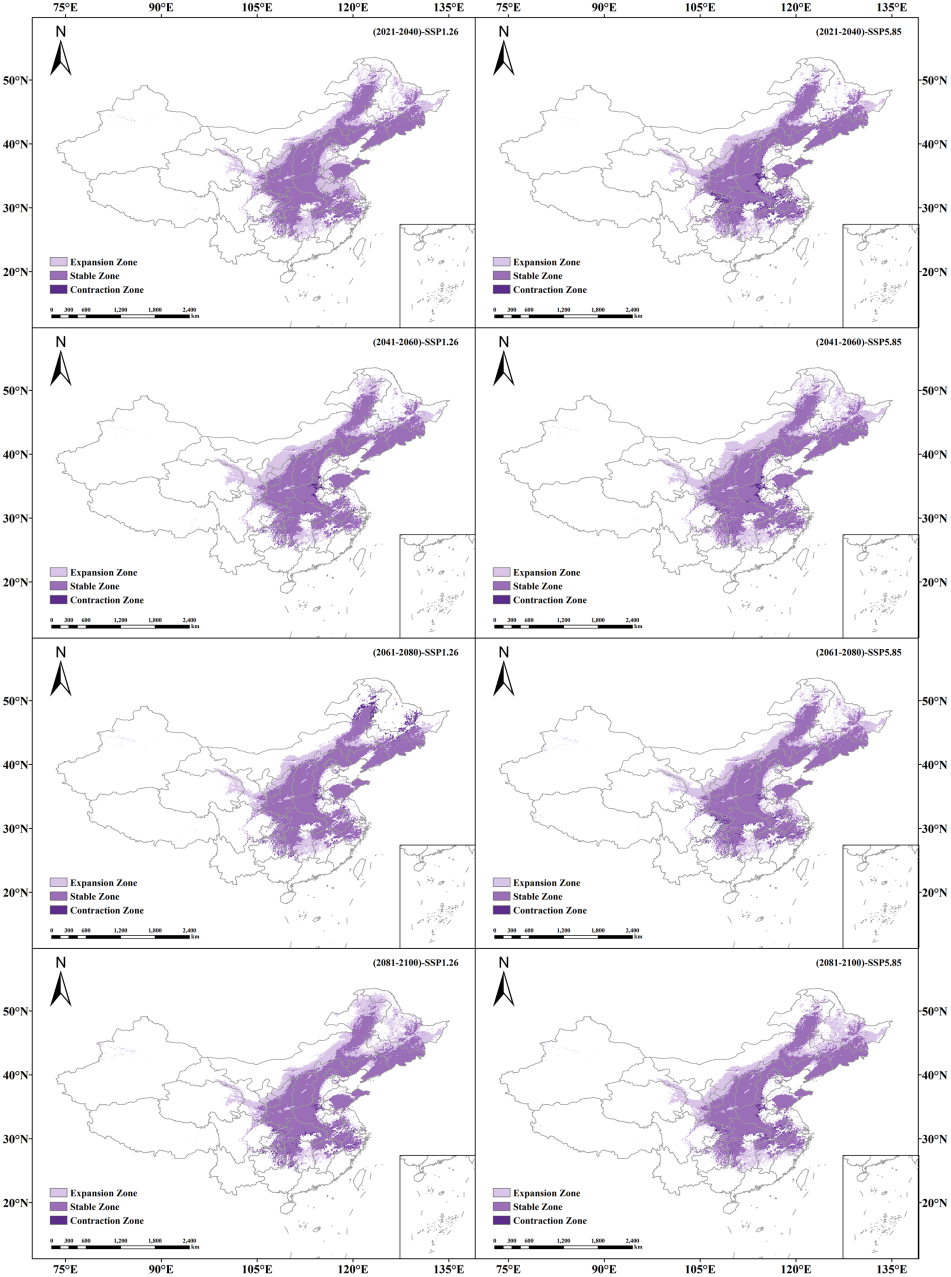
Spatial pattern changes in the potential suitable habitats of *A. lancea* under future climate scenarios.

**Figure 10:**
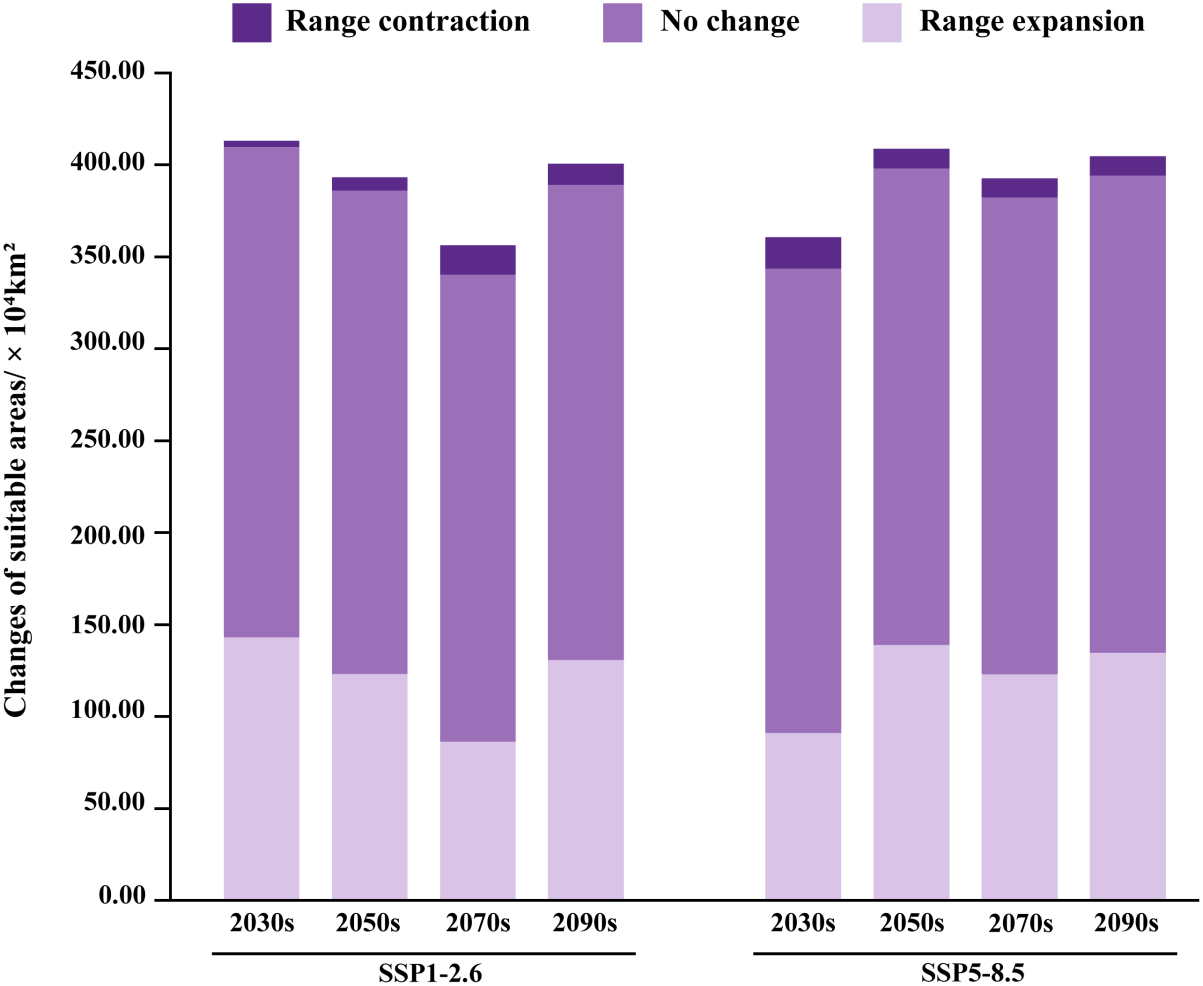
Overlay of changes in the spatial pattern of potential suitable habitats for *A. lancea* under future climate scenarios.

Spatial overlay of the future suitable surfaces with the present-day surface partitioned the change in *A. lancea* habitat into three classes: expansion areas, contraction areas, and retention areas (**Figures 9**, **10**).

Under SSP1-2.6, spatial change in the suitable habitat of *A. lancea* is comparatively pronounced. Expansion areas push markedly toward higher latitudes, peaking at 1.43 × 10^6^ km^2^ in the 2030s, with the new suitable land concentrated in southern Inner Mongolia, eastern Hunan and the central part of North China; contraction areas grow steadily through time and reach 1.16 × 10^5^ km^2^ by the 2090s, occurring mainly along the southern margin of the traditional *A. lancea* range — central Henan, eastern Inner Mongolia, eastern Guizhou and southern Jiangsu. Retention areas remain dominant overall but shrink slightly through time. Taken as a whole, SSP1-2.6 produces a “southward contraction – northward expansion” pattern of *A. lancea* habitat change.

Under SSP5-8.5, spatial change in the suitable habitat of *A. lancea* is comparatively gradual. Expansion areas are mainly distributed along the northern and northwestern margins of the present-day habitat — southern Gansu, southern Ningxia, northern Hebei, southeastern Inner Mongolia and eastern Hunan — with the expansion area increasing from 9.10 × 10^5^ km^2^ in the 2030s to 1.35 × 10^6^ km^2^ in the 2090s. Contraction areas remain comparatively stable, fluctuating between 1.00 × 10^5^ km^2^ and 1.70 × 10^5^ km^2^ across periods, and are concentrated along the southern margin of the traditional habitat (northern Henan, southern Hubei, southern Anhui and parts of southern Jiangsu). Retention areas remain dominant throughout, varying between 2.50 × 10^6^ km^2^ and 2.60 × 10^6^ km^2^.

### 3.7 Centroid migration of suitable habitats under future climate scenarios

**Figure 11:**
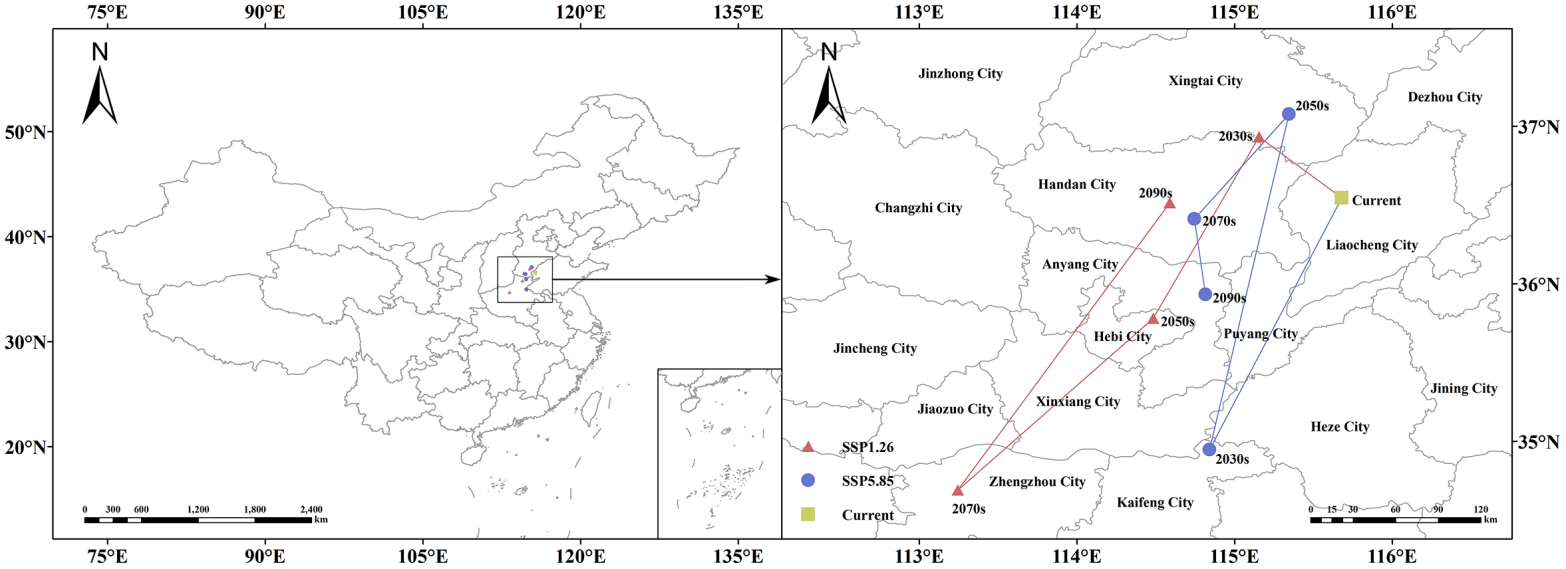
Migration trajectory of the geographic centroid of suitable areas of *A. lancea* under future climate scenarios.

Using MTSPS = 0.2727 as the threshold and the Zonal Geometry tool in ArcGIS, the geographic centroids of suitable habitat under the present climate and each future scenario were calculated, and migration trajectories were mapped (**Figure 11**). Under the present climate, the geographic centroid was located in Guan County, Liaocheng City, Shandong Province (115.44°E, 36.48°N).

Under SSP1-2.6 the centroid follows a relatively volatile trajectory. Between 2021 and 2040 it shifts 63.56 km north-westward to Guangzong County, Xingtai City, Hebei Province (115.14°E, 37.07°N); between 2041 and 2060 it then moves 141.83 km south-westward to Xun County, Hebi City, Henan Province (114.55°E, 35.67°N); between 2061 and 2080 it continues 166.83 km south-westward to Gongyi City, Zhengzhou City, Henan Province (113.02°E, 34.75°N); and between 2081 and 2100 it reverses and travels 237.61 km north-eastward, ending in Hanshan District, Handan City, Hebei Province (114.49°E, 36.58°N).

Under SSP5-8.5 the centroid of the suitable habitat of *A. lancea* likewise shows pronounced spatial fluctuation. Between 2021 and 2040 it first moves 192.62 km south-westward to Lankao County, Kaifeng City, Henan Province (114.82°E, 34.82°N); between 2041 and 2060 it reverses 240.38 km north-eastward to Wei County, Xingtai City, Hebei Province (115.27°E, 36.97°N); between 2061 and 2080 it shifts 90.91 km south-westward to Cheng’an County, Handan City, Hebei Province (114.67°E, 36.44°N); and between 2081 and 2100 it travels 53.06 km southward to Neihuang County, Anyang City, Henan Province (114.90°E, 35.97°N).

Across both pathways, centroid locations fluctuated among western Shandong, southern Hebei, and northern Henan. The direction and magnitude of migration varied among periods and scenarios, rather than following a uniform poleward trajectory.

## 4 Discussion

Building on the data cleaning and variable screening described above, the present work further tuned MaxEnt parameters to improve prediction stability. Occurrence records were thinned on a 5 km × 5 km grid to reduce spatial autocorrelation, and the 90 candidate variables were screened by combining Pearson correlation analysis, ecological interpretability, auxiliary contribution information, and VIF verification. The final eight predictors had VIF values below 5, indicating no severe multicollinearity. The optimized parameter combination, FC = LH and RM = 3, reduced the omission rate from 5.4% to 1.8% and lowered AICc relative to the default model while retaining good discrimination against background locations (mean training AUC = 0.923). Rather than representing a new modelling algorithm, this tuning step provided a more parsimonious species-specific parameterization for subsequent projections.

The results identify July precipitation (prec_07) and temperature seasonality (bio_4) as the two leading climatic determinants of the distribution of *A. lancea* in China, jointly accounting for 61.7% of the modelled signal. This finding meshes well with the species’ biology: July is the rainfall-concentration month over the East Asian monsoon belt and also the period during which rhizome biomass and volatile-oil secondary metabolites accumulate most actively. The response curve indicates a 129–229 mm optimum window for July precipitation — too little rainfall fails to meet the water demand for growth, while too much triggers root waterlogging and rhizome-rot diseases, both consistent with the ecological description of *A. lancea* as preferring cool, humid conditions and intolerant of high temperatures and water-logged soils (Jun et al., 2018; Koonrungsesomboon et al., 2014). Temperature seasonality (bio_4), an index of intra-annual temperature amplitude, has an optimum of 834–1275 (°C × 100), placing the species in warm-temperate climates with marked winter-summer contrasts. A moderate seasonal swing favors both the dormancy-germination cycle of the rhizomes and the build-up of bioactive constituents; tropical climates with little seasonality, and extreme continental climates with very large amplitudes, both fall outside the suitable envelope. In addition, the comparatively high contributions of altitude (16.0%) and vegetation subtype (14.7%) imply that topography and vegetation, by mediating the local redistribution of moisture and heat, also play a meaningful role in shaping the spatial pattern of *A. lancea*.

Under the present climate, the suitable habitat of *A. lancea* covers about 2.72 × 10^6^ km^2^ and is concentrated in the warm-temperate monsoon belt of northern China, with the high-suitability core in western Liaoning, the northern Shandong Peninsula, the northern North China Plain, and northern Henan, broadly matching the core distribution of *A. lancea* recorded in Flora of China. The historical Dao-di (authentic provenance) production regions of *A. lancea* are concentrated mainly in the Maoshan area of Jiangsu and the Dabie Mountains of Anhui, while modern cultivation has expanded to Chifeng in Inner Mongolia, Chengde in Hebei, Luotian in Hubei, and mountainous areas of southern Anhui. Previous quality-regionalization and geographic-origin studies have shown that ecological suitability, chemical quality, and producing-region identity do not always coincide exactly (Zhu et al., 2017; Wang et al., 2023a; Zhang et al., 2023). This supports the interpretation that predicted suitability is an ecological reference rather than a direct substitute for Dao-di medicinal-material quality evaluation.

The county-level overlay analysis showed both consistency and divergence between traditional or reported producing regions of *A. lancea* and predicted current suitable habitats. Several producing regions in Hubei and Henan overlapped strongly with predicted suitable habitats, suggesting that the optimized MaxEnt model can identify areas that are environmentally suitable for *A. lancea* under current climatic and environmental conditions. These results provide a spatial reference for modern resource conservation and cultivation planning.

However, some Maoshan-related traditional producing regions showed relatively weak current suitability in the model. This indicates that Dao-di medicinal-material production regions cannot be explained simply by present-day climatic suitability. The formation of geoherbal medicinal materials is usually influenced by natural environments, historical distribution, long-term cultivation, harvesting and processing practices, commodity circulation, and quality evaluation systems. For *A. lancea*, the geoherbal significance of Maoshan-related regions may depend not only on broad-scale current climate, but also on local habitats, cultivation and harvesting traditions, accumulation of medicinal constituents, and historical recognition. Therefore, ecological niche model predictions should be interpreted as an environmental-suitability reference rather than as a direct confirmation or rejection of the value of traditional Dao-di medicinal-material production regions.

The pattern of partial consistency and partial divergence also suggests that future studies of *A. lancea* resources should integrate ecological niche modelling with geoherbal quality evaluation, population genetics, medicinal-quality formation, and land-use change. Only by considering both ecological suitability and medicinal-quality-related factors can resource conservation, standardized cultivation, and geoherbal producing-region management of *A. lancea* be more accurately supported.

The future projections show clear changes in the spatial pattern of *A. lancea*. Under both pathways, expansion was concentrated mainly along the northern and northwestern margins of the present-day habitat, whereas contraction occurred primarily along parts of the southern margin; correspondingly, the total suitable area was larger than the current baseline in all projected periods. This pattern is consistent with the tendency for warming to redistribute plant ranges toward higher latitudes (Chen et al., 2011; Lenoir et al., 2008). Studies of other medicinal plants, including *Leonurus japonicus*, *Angelica dahurica*, and *Panax notoginseng*, have likewise reported scenario-dependent expansion, contraction, or displacement of suitable areas under future climates (Wang et al., 2023b; Zhang et al., 2024; Zhan et al., 2022). In the present study, centroid locations moved repeatedly among western Shandong, southern Hebei, and northern Henan, and the direction and distance of movement differed between scenarios and periods. These reversals show that centroid migration was not monotonic and should be interpreted as scenario-dependent spatial redistribution rather than a continuous northward shift. The projected changes provide a spatial reference for future field surveys, conservation planning, and cautious evaluation of potential cultivation areas.

Although the MaxEnt model used here was systematically optimized through occurrence filtering, variable screening, VIF verification, and parameter tuning, several sources of uncertainty remain. First, spatial thinning reduced local clustering of occurrence records, but it did not eliminate sampling bias in herbarium and public-database records. Because a bias file, target-group background, or explicit spatial bias-correction layer was not used, areas with denser historical collecting effort may still influence model calibration. Second, the retained variables were selected using correlation analysis, ecological interpretation, auxiliary contribution information, and VIF verification, but alternative correlation thresholds or a strictly correlation-first/ecology-first strategy could produce a different predictor set. Third, the threshold-dependent TSS calculated at the MTSPS threshold provides an additional quantitative reference for evaluating the selected model. However, Boyce or continuous Boyce indices, fully independent absence/background validation data, and spatial block cross-validation were not incorporated, so spatial transferability and threshold-independent performance remain incompletely quantified. Fourth, only one species distribution modelling algorithm and one GCM were used. Multi-model SDM ensembles and multiple GCMs would better represent structural and climate-model uncertainty, especially for future projections. Fifth, land-use change, human harvesting pressure, cultivation management, accessibility, conservation policy, and medicinal-quality indicators were not included in the present model. Therefore, the predicted areas represent potential ecological suitability and should not be equated with actual resource abundance, directly usable cultivation land, or geoherbal quality. Finally, the overlay with traditional or reported producing regions was conducted at the county/district scale, and township- or site-level quality differences were not identified. Future research should combine field investigation, medicinal-quality evaluation, local environmental data, land-use scenarios, spatially explicit validation, and multi-GCM ensemble projections to clarify the relationships among suitable distribution, geoherbal quality formation, and climate-change responses in *A. lancea*.

## 5 Conclusion

Taking *A. lancea* across China as the focal species, this study used an optimized MaxEnt model to predict its current and future potential suitable habitats and identify key environmental drivers. July precipitation and temperature seasonality were the two dominant climatic factors, while altitude and vegetation subtype also contributed substantially. Under current conditions, suitable habitats were concentrated mainly in northern, central, and eastern China, with a total suitable area of approximately 2.72 × 10^6^ km^2^. Under SSP1-2.6 and SSP5-8.5, the total suitable area generally increased, and geographic centroid locations fluctuated among western Shandong, southern Hebei, and northern Henan, with distinct trajectories under the two scenarios.

The county/district-level overlay analysis showed that some modern or reported producing regions overlapped well with predicted current suitability, whereas some Maoshan-related traditional producing regions did not fully match the current climatic-suitability pattern. These findings suggest that predicted suitable areas should be interpreted as potential ecological suitability, not as actual cultivation/resource suitability or direct proof of geoherbal quality. Because future projections were based on the selected GCM and did not include dynamic land use, human disturbance, or medicinal-quality indicators, the results should be used as a spatial reference rather than as a complete production-suitability assessment. Future studies should integrate multi-model and multi-GCM ensemble prediction, land-use and human-disturbance factors, spatially explicit validation, field investigation, and medicinal-quality evaluation to better support conservation, cultivation planning, and geoherbal producing-region management of *A. lancea*.

## Data Availability

The raw data supporting the conclusions of this article will be made available by the authors, without undue reservation.

## References

[B1] Anderson R. P. (2013). A framework for using niche models to estimate impacts of climate change on species distributions. Ann. N. Y. Acad. Sci. 1297, 8–28. doi: 10.1111/nyas.12264 25098379

[B2] Applequist W. L. Brinckmann J. A. Cunningham A. B. Hart R. E. Heinrich M. Katerere D. R. . (2020). Scientists’ warning on climate change and medicinal plants. Planta Med. 86, 10–18. doi: 10.1055/a-1041-3406 31731314

[B3] Bailly C. (2021). Atractylenolides, essential components of Atractylodes-based traditional herbal medicines: antioxidant, anti-inflammatory and anticancer properties. Eur. J. Pharmacol. 891, 173735. doi: 10.1016/j.ejphar.2020.173735 33220271

[B4] Boria R. A. Olson L. E. Goodman S. M. Anderson R. P. (2014). Spatial filtering to reduce sampling bias can improve the performance of ecological niche models. Ecol. Model. 275, 73–77. doi: 10.1016/j.ecolmodel.2013.12.012 38826717

[B5] Chen I.-C. Hill J. K. Ohlemüller R. Roy D. B. Thomas C. D. (2011). Rapid range shifts of species associated with high levels of climate warming. Science 333, 1024–1026. doi: 10.1126/science.1206432 21852500

[B6] Cobos M. E. Peterson A. T. Barve N. Osorio-Olvera L. (2019). kuenm: an R package for detailed development of ecological niche models using Maxent. PeerJ 7, e6281. doi: 10.7717/peerj.6281 30755826 PMC6368831

[B7] Dormann C. F. Elith J. Bacher S. Buchmann C. Carl G. Carré G. . (2013). Collinearity: a review of methods to deal with it and a simulation study evaluating their performance. Ecography 36, 27–46. doi: 10.1111/j.1600-0587.2012.07348.x 40046247

[B8] Elith J. Phillips S. J. Hastie T. Dudík M. Chee Y. E. Yates C. J. (2011). A statistical explanation of MaxEnt for ecologists. Divers. Distrib. 17, 43–57. doi: 10.1111/j.1472-4642.2010.00725.x 40046247

[B9] Eyring V. Bony S. Meehl G. A. Senior C. A. Stevens B. Stouffer R. J. . (2016). Overview of the Coupled Model Intercomparison Project Phase 6 (CMIP6) experimental design and organization. Geosci. Model. Dev. 9, 1937–1958. doi: 10.5194/gmd-9-1937-2016

[B10] Hijmans R. J. Cameron S. E. Parra J. L. Jones P. G. Jarvis A. (2005). Very high resolution interpolated climate surfaces for global land areas. Int. J. Climatol. 25, 1965–1978. doi: 10.1002/joc.1276 41531421

[B11] Jun X. Fu P. Lei Y. Cheng P. (2018). Pharmacological effects of medicinal components of Atractylodes lancea (Thunb.) DC. Chin. Med. 13, 59. doi: 10.1186/s13020-018-0216-7 30505341 PMC6260578

[B12] Koonrungsesomboon N. Na-Bangchang K. Karbwang J. (2014). Therapeutic potential and pharmacological activities of Atractylodes lancea (Thunb.) DC. Asian Pac. J. Trop. Med. 7, 421–428. doi: 10.1016/S1995-7645(14)60069-9 25066389

[B13] Lenoir J. Gégout J. C. Marquet P. A. de Ruffray P. Brisse H. (2008). A significant upward shift in plant species optimum elevation during the 20th century. Science 320, 1768–1771. doi: 10.1126/science.1156831 18583610

[B14] Liu C. Berry P. M. Dawson T. P. Pearson R. G. (2005). Selecting thresholds of occurrence in the prediction of species distributions. Ecography 28, 385–393. doi: 10.1111/j.0906-7590.2005.03957.x 40046247

[B15] Merow C. Smith M. J. Silander J. A. (2013). A practical guide to MaxEnt for modeling species’ distributions: what it does, and why inputs and settings matter. Ecography 36, 1058–1069. doi: 10.1111/j.1600-0587.2013.07872.x 40046247

[B16] Muscarella R. Galante P. J. Soley-Guardia M. Boria R. A. Kass J. M. Uriarte M. . (2014). ENMeval: an R package for conducting spatially independent evaluations and estimating optimal model complexity for Maxent ecological niche models. Methods Ecol. Evol. 5, 1198–1205. doi: 10.1111/2041-210X.12261 40046247

[B17] Phillips S. J. Anderson R. P. Schapire R. E. (2006). Maximum entropy modeling of species geographic distributions. Ecol. Model. 190, 231–259. doi: 10.1016/j.ecolmodel.2005.03.026 38826717

[B18] Rana S. K. Rana H. K. Ranjitkar S. Ghimire S. K. Gurmachhan C. M. O’Neill A. R. . (2020). Climate-change threats to distribution, habitats, sustainability, and conservation of highly traded medicinal and aromatic plants in Nepal. Ecol. Indic. 115, 106435. doi: 10.1016/j.ecolind.2020.106435 38826717

[B19] Riahi K. van Vuuren D. P. Kriegler E. Edmonds J. O’Neill B. C. Fujimori S. . (2017). The Shared Socioeconomic Pathways and their energy, land use, and greenhouse gas emissions implications: an overview. Glob. Environ. Change 42, 153–168. doi: 10.1016/j.gloenvcha.2016.05.009 38826717

[B20] Wang H. Y. Ma W. Q. Jing Z. X. Jiang D. Q. Peng Z. Xu Y. . (2023a). Ecological suitability and quality regionalization of Atractylodes lancea based on MaxEnt and GIS. World Chin. Med. 18, 1847–1856. doi: 10.3969/j.issn.1673-7202.2023.13.010

[B21] Wang Y. Xie L. Zhou X. Chen R. Zhao G. Zhang F. (2023b). Prediction of the potentially suitable areas of Leonurus japonicus in China based on future climate change using the optimized MaxEnt model. Ecol. Evol. 13, e10597. doi: 10.1002/ece3.10597 37869439 PMC10585429

[B22] Warren D. L. Seifert S. N. (2011). Ecological niche modeling in MaxEnt: the importance of model complexity and the performance of model selection criteria. Ecol. Appl. 21, 335–342. doi: 10.1890/10-1171.1 21563566

[B23] Wu T. Lu Y. Fang Y. Xin X. Li L. Li W. . (2019). The Beijing Climate Center Climate System Model (BCC-CSM): the main progress from CMIP5 to CMIP6. Geosci. Model. Dev. 12, 1573–1600. doi: 10.5194/gmd-12-1573-2019

[B24] Wu Z. Y. Raven P. H. Hong D. Y. (2011). Flora of China, Vol. 20–21 (Asteraceae). Eds. Wu Z. Y. Raven P. H. Hong D. Y. (Beijing/St. Louis: Science Press/Missouri Botanical Garden Press).

[B25] Yang J. Jiang P. Huang Y. Yang Y. Wang R. Yang Y. (2022). Potential geographic distribution of relict plant Pteroceltis tatarinowii in China under climate change scenarios. PloS One 17, e0266133. doi: 10.1371/journal.pone.0266133 35395025 PMC8993005

[B26] Yang X. Q. Kushwaha S. P. S. Saran S. Xu J. Roy P. S. (2013). MaxEnt modeling for predicting the potential distribution of medicinal plant, Justicia adhatoda L. in Lesser Himalayan foothills. Ecol. Eng. 51, 83–87. doi: 10.1016/j.ecoleng.2012.12.004 38826717

[B27] Zhan P. Wang F. Xia P. Zhao G. Wei M. Wei F. . (2022). Assessment of suitable cultivation region for Panax notoginseng under different climatic conditions using MaxEnt model and high-performance liquid chromatography in China. Ind. Crops Prod. 176, 114416. doi: 10.1016/j.indcrop.2021.114416 38826717

[B28] Zhang C. Wang H. Lyu C. Wang Y. Sun J. Zhang Y. . (2023). Authenticating the geographic origins of Atractylodes lancea rhizome chemotypes in China through metabolite marker identification. Front. Plant Sci. 14, 1237800. doi: 10.3389/fpls.2023.1237800 37841605 PMC10569125

[B29] Zhang F.-G. Liang F. Wu K. Xie L. Zhao G. Wang Y. (2024). The potential habitat of Angelica dahurica in China under climate change scenario predicted by MaxEnt model. Front. Plant Sci. 15, 1388099. doi: 10.3389/fpls.2024.1388099 39135644 PMC11317415

[B30] Zhu S. Peng H. Guo L. Xu T. Zhang Y. Chen M. . (2017). Regionalization of Chinese material medical quality based on maximum entropy model: a case study of Atractylodes lancea. Sci. Rep. 7, 42417. doi: 10.1038/srep42417 28205539 PMC5311955

