# Supplementary material for: Predicting the potential distribution of Atractylodes lancea in China under CMIP6 climate scenarios using an optimized MaxEnt model
Source: Front Plant Sci. 2026 Aug 3;17:1881018. doi: 10.3389/fpls.2026.1881018 (PMC13478382; doi:10.3389/fpls.2026.1881018)
Supplement: Supplementary file 1 [file DataSheet7.pdf]

County-level producing regions and current predicted suitability of *Atractylodes lancea*

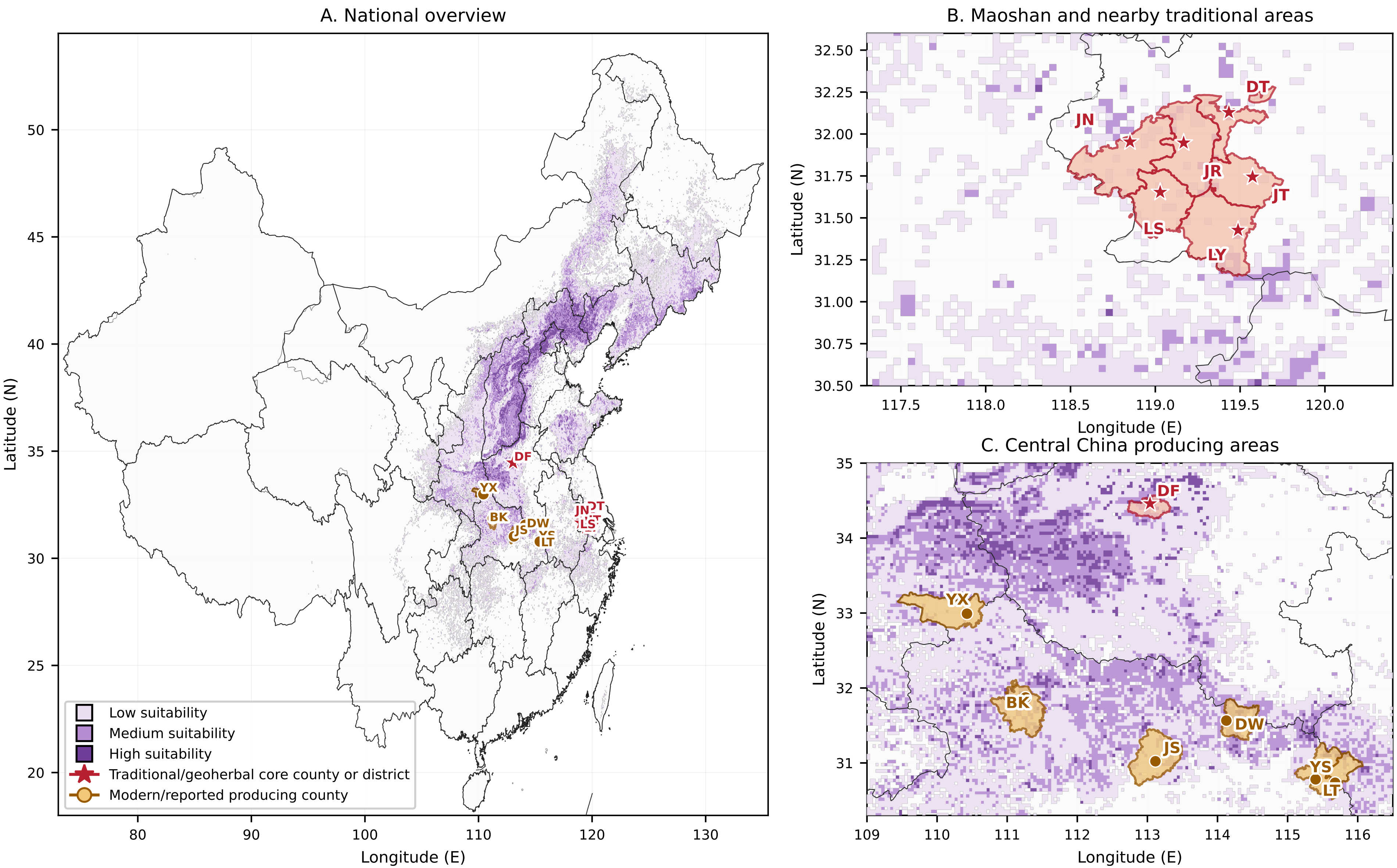

Codes: JR Jurong; DT Dantu; JN Jiangning; JT Jintan; LY Liyang; LS Lishui;
DF Dengfeng; YS Yingshan; LT Luotian; YX Yunxi; BK Baokang; JS Jingshan; DW Dawu.
